# Exploration of factors influencing the preservation of residual hearing following cochlear implantation

**DOI:** 10.4102/sajcd.v66i1.607

**Published:** 2019-04-10

**Authors:** Katijah Khoza-Shangase, Katherine Gautschi-Mills

**Affiliations:** 1 Department of Speech Pathology and Audiology, University of the Witwatersrand, Johannesburg, South Africa

**Keywords:** Cochlear implant, complications, electrode, intracochlear, preservation, residual hearing

## Abstract

**Background:**

Increasing access to cochlear implants within the resource-constrained South African context calls for careful investigation of all factors that might influence benefit from this technological advancement.

**Objective:**

The aim of this study was to investigate preservation of hearing following cochlear implant surgery and whether a relationship existed between the post-operative hearing findings and certain factors.

**Methods:**

Within a quantitative paradigm, a retrospective data review design was adopted where a sample consisting of audiological records from 60 observations and surgical records from two cochlear implant units in South Africa was investigated. These records were selected using purposive sampling and consisted of records from participants ranging from 6 to 59 years. Comparative analysis of unaided audiological test results was pre- and post-operatively performed, where all paitents were implanted with cochlear devices. Factors documented to have a possible influence on post-operative outcomes were examined in an attempt to establish relationships that may exist. Findings were analysed by means of both inferential and descriptive statistics.

**Results:**

The findings indicated 92% success rate in preservation of residual hearing. There was a direct correlation between surgical techniques, as well as cochlear implant type and the successful hearing findings, in the absence of surgical complications. Other factors explored did not have any negative effect on the hearing findings.

**Conclusion:**

The study findings suggest improved surgical outcomes with enhanced surgical techniques and advanced technology, with a clear negative impact of intraoperative complications on the outcomes.

## Background

Evidence of improved speech perception because of technological advances in cochlear implantation and improvements in surgical techniques exists (Balkany et al., 2006), and this includes patients with residual hearing in low frequencies. Historically, patients with residual hearing were excluded from implantation even though they experienced poor speech discrimination when fitted with high-quality digital hearing aids (Skarzynski et al., 2002). Furthermore, historically, there was a belief that residual hearing was lost during cochlear implantation as a result of trauma caused by the insertion of the electrode array (Balkany et al., 2006).This is, however, no longer the held belief as enough evidence has shown preserved residual hearing post-implantation. This residual of hearing post-implantation has facilitated the introduction of bimodal electro-acoustic stimulation (EAS), which involves using a combination of cochlear implant and hearing aid use in the same ear (Balkany et al., 2006). This evidence highlights the importance of complication minimal surgical techniques.

Success in conservation of residual hearing after cochlear implantation has benefited patients with high levels of residual low-frequency hearing who were not previously considered for conventional cochlear implantation (Skarzynski et al., 2002), hence the importance of exploring possible influencing factors in the success or failure of preserving residual hearing during this process. Although some implantees lose their residual hearing post-surgery despite all efforts to preserve it, the rate at which implantees have their residual hearing preserved is reportedly increasing at a steady pace. One of the cited reasons for this is the expanding knowledge and experience of advancements in electrode designs and surgical techniques, resulting in minimal trauma (Gstoettner et al., 2009). Di Nardo et al. (2007) found that it was possible to have minimal trauma to structures in the cochlea, following electrode array insertion during cochlear implant surgery.

Skarzynski et al. (2002) investigated the preservation of residual hearing using the ‘soft surgery’ technique and atraumatic electrode insertion and found that 62% of implantees retained their residual hearing and 19% experienced a total loss of functional hearing. Furthermore, they found that chronological age, gender, aetiology and implant type did not have an influence on the hearing findings – these results are consistent with those by Cosetti et al. (2013). Such findings highlight the importance of investigating factors that might have a possible influence on the preservation of residual hearing following cochlear implants; hence, the current study was undertaken within the South African context.

## Methods

### Main aim

This study formed part of a study titled ‘Preservation of residual hearing after cochlear implant surgery: An exploration of residual hearing function in a group of recipients at cochlear implant units’ (Gautschi-Mills, Khoza-Shangase, & Pillay, 2018). The primary objective was to explore the preservation of residual hearing function in a group of cochlear implant recipients in two cochlear implant units in South Africa. The study’s specific secondary aim was to establish whether a relationship exists between the hearing findings and the following factors:

- degree of preoperative hearing loss
- aetiologies of hearing loss
- duration of hearing loss prior to surgery
- duration between surgery and unaided post-operative hearing testing
- electrode type
- electrode array insertion
- electrode array insertion depth
- surgical techniques
- intraoperative complications.

### Research design

This study adopted a quantitative design in which a retrospective data review was conducted. Existing pre- and post-operative unaided audiological testing results as well as surgical records of patients who had cochlear implant surgery were reviewed to establish whether there was a relationship between the hearing findings and the various factors.

A total of 53 audiological records and 50 surgical records were included in the study. Although there were 53 participants, seven of these individuals were bilaterally implanted, yielding a sample size of 60 observations. The majority of the participants (64%) were women, with the mean age being 30.8 years. All participant records had to meet specific inclusion criteria. Participation criteria used in South Africa include cochlear implant fitting, residual preoperative hearing thresholds, less than 60% for sentence recognition in the ear to be implanted (Wagenfeld et al., 2004) and intact auditory nerve functioning (with the absence of auditory neuropathy) (Moctezuma & Tu, 2011) were adopted.

An adapted surgical form, originally devised by one of the units, was completed, using surgical notes from participant files:

- The researcher collected additional data, which included the following:
  ▪ age, gender, aetiology, duration of hearing loss prior to implantation (in years and months), electrode type, electrode array insertion, electrode array depth, surgical technique and intraoperative complications.

### Data analysis

The change in hearing thresholds from preoperative to post-operative hearing threshold levels was classified for each participant, with a change of 0–10 dB depicting complete preservation of residual hearing, a change greater than 10 dB indicating partial preservation and no response post-surgery indicating complete loss of residual hearing. These findings are published by Gautschi-Mills et al. (2018), and the readers are referred there for further details.

Pre- and post-operative unaided thresholds were presented in order to compare them. For inferential statistics, the 95% confidence level was used throughout. General patterns or trends were examined to determine whether certain relationships existed between the post-operative hearing thresholds and the following:

- degree of preoperative hearing
- duration between surgery and unaided post-operative hearing testing
- electrode type.

The *X*^2^ test was used to assess the relationships between categorical variables.

## Ethical consideration

Ethical clearance was obtained prior to the commencement of the study (Protocol number: M111037), with permission from all relevant authorities. Furthermore, this research adhered to the Helsinki Declaration of 1975, as revised in 2008.

## Results and discussion

### Hearing thresholds before and after surgery

**FIGURE 1:**
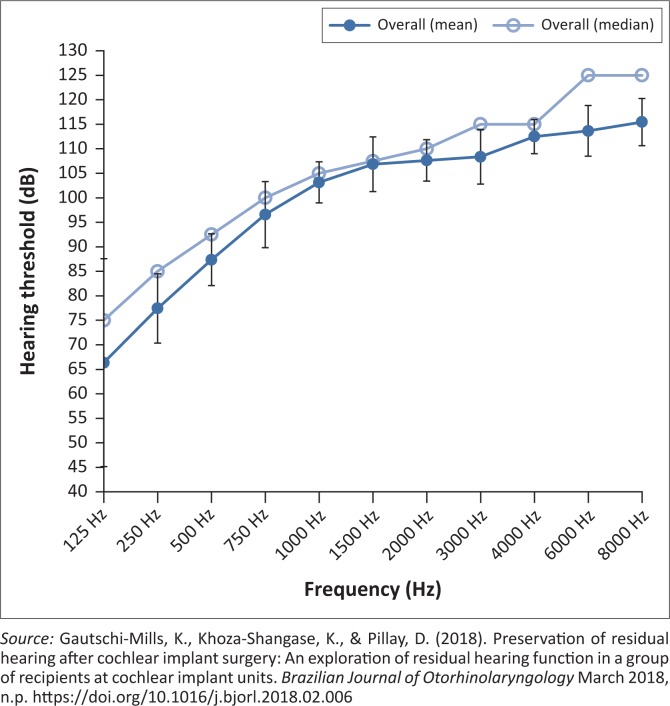
Preoperative hearing threshold levels overall.

**FIGURE 2:**
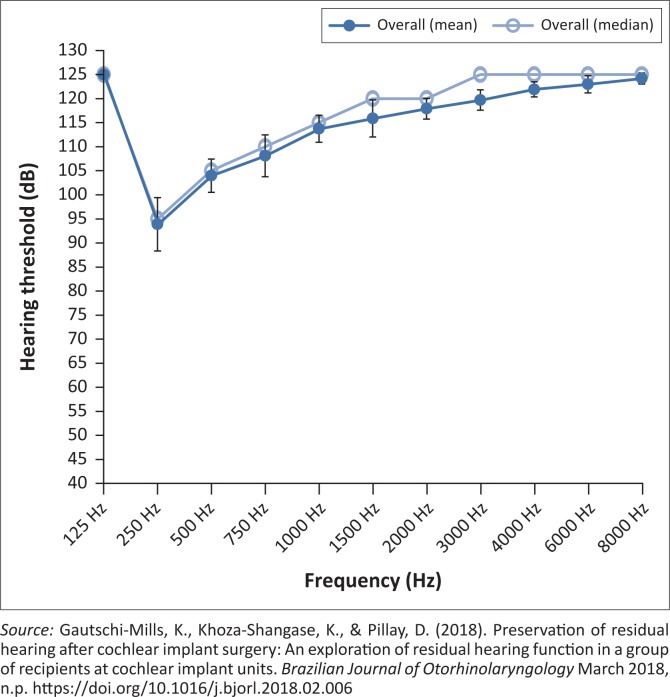
Post-operative hearing threshold levels overall.

Findings from the study of which this current study formed a part, revealed that preoperatively participants in the overall sample presented with severe to profound range of hearing loss, with some degree of low-frequency residual hearing with poorer hearing in the high frequencies (Figures 1 and 2) (Gautschi-Mills et al., 2018).

These findings are consistent with the tonotopicity of the cochlea, where high frequencies are more susceptible to damage than lower frequencies (Martin & Clark, 2006). Post-operatively, the results obtained indicated a high-frequency loss, with some preservation of the low frequencies and slight preservation of the mid-frequencies post-operatively. Although the results indicated some preservation of low-frequency and mid-frequency hearing, it was clear that there was some loss in residual hearing, which means that the hearing threshold levels before surgery dropped further after surgery (Gautschi-Mills et al., 2018).

Overall, 92% of the implantees experienced hearing preservation post-operatively – either partial or complete hearing preservation. These findings partly supported previous findings by Skarzynski et al. (2002) and Cosetti et al. (2013) who found hearing preservation post-operatively under similar conditions as in the current study.

### The relationship between the hearing findings and various factors

#### Degree of preoperative hearing loss

Current findings indicated that the degree of preoperative low-frequency hearing had no effect on the post-operative hearing findings. Similarly, Cosetti et al. (2013) found that there was no significant relationship between the low-frequency pure-tone average and the post-operative hearing findings.

Current findings are inconsistent with those by Balkany et al. (2006) who found that there was a relationship between the degree of preoperative hearing loss and the hearing findings, in that those patients who had a greater degree of preoperative hearing loss tended to experience a complete loss of residual hearing post-operatively. In the current study, there was no significant correlation between the preoperative hearing threshold levels and the preservation of post-operative residual hearing (*p* = 0.174).

Despite the fact that there was a correlation between the degree of preoperative hearing threshold levels and post-operative hearing in Balkany et al.’s (2006) study, this was limited to patients who experienced a complete loss of residual hearing post-operatively. In general, there was no significant relationship between the degree of preoperative hearing loss and the unaided post-operative hearing findings.

### Aetiologies of hearing loss

As depicted in Table 1, the main categories for aetiologies of hearing loss varied, with congenital causes being the most commonly occurring aetiology. Congenital causes of hearing impairment were also found in Derinsu, Serin, Akdas and Batman’s (2011) study, where the aetiologies of hearing loss indicated a predominance of congenital factors.

**TABLE 1:** Aetiologies of hearing loss found in the current study.

Aetiology	Overall %
Congenital	38
Disease or virus	37
Progressive	10
Damage to cochlea	5
Other	3
Missing	27

An analysis of the relationship between the hearing findings and aetiology revealed that the effect of aetiology on change in hearing thresholds was not significant for any of the frequencies. The fact that aetiologies in the present study did not have any effect on the hearing findings is consistent with the findings of Skarzynski et al. (2002) who found that aetiologies had no influence on the post-operative hearing findings.

#### Duration of hearing loss prior to surgery

The mean duration of hearing loss prior to surgery was established to determine the relationship between the duration of the hearing loss before surgery and the post-operative hearing findings.

The study findings indicated that the mean duration of hearing loss prior to surgery was 22 years and 9 months (s.d. = ± 3.1) overall. The effect of duration of hearing loss was not significant for any of the frequencies. Thus, there was no significant difference in terms of post-operative hearing threshold levels between those implantees who had a longer duration of preoperative hearing loss and those implantees who experienced hearing loss for a shorter period of time. This is contrary to what Lenarz et al. (2009) found, that patients with a shorter duration of preoperative hearing loss had better hearing preservation post-operatively than those with a longer duration of loss preoperatively. Current findings are consistent with those of Cosetti et al. (2013) who found that there was no correlation between the duration of hearing loss and the unaided post-operative hearing threshold findings.

#### Duration between surgery and unaided post-operative hearing testing

The researcher examined the mean time between surgery and first post-operative hearing test to ascertain whether this had had any influence on the post-operative hearing threshold levels. This was used as a covariate in the analysis of the results in order to prevent the findings being affected by any extraneous variables. The mean time between surgery and first post-operative hearing test was 24.7 months (s.d. = ±9.0) (range 0.5–159 months).

Findings on the relationship between the hearing findings and the duration between surgery and unaided post-operative hearing testing indicated that this time was not significant. This means that the duration between surgery and unaided post-operative hearing testing did not affect the preservation of residual hearing post-operatively. This is a notable finding from this study where the upper extreme was 13 years and 4 months. These findings seem contrary to previously published research where a number of patients lost their residual hearing at different times post-surgery, with 30% of the sample experiencing a low-frequency threshold drop of more than 30 dB over time (Gantz et al., 2009).

#### Electrode type

As shown in Table 2, the category ‘Other’ (17%) included *inter alia* the electrode type –Nucleus CI24R with Contour Advance electrode (*n* = 5). In this study, the electrode type that was used most after the two categories mentioned above (namely, the CI512 and the CI24RE) was the CI24R. However, there were only five observations for the CI24R. There were even fewer observations for other electrode types that included CI24M, Nucleus CI24(ST) and Nucleus CI422. Statistical analysis could not be performed on these electrode types as there was not sufficient data because the number of observations was too small.

**TABLE 2:** Electrode types for participants in the current study (*N* = 60).

Electrode type	Overall %
NF – CI24RE	50
CN – CI512	25
Other	17
Missing	8

NF – CI24RE, Nucleus Freedom Cochlear Implant with Contour Advance or Straight electrodes; CI512, Nucleus CI512 Cochlear Implant with Contour Advance electrode.

The effect of electrode type on change in hearing thresholds was not significant for any of the frequencies, with the exception of 2000 Hz: the mean change in hearing thresholds was 5.9 dB higher for other versus Nucleus Freedom (NF). In other words, the electrode type did not have a negative effect on the post-operative hearing threshold levels (or the change in residual hearing), with the exception of that at 2000 Hz. Given that there was no trend with respect to the other frequencies, this could have been a spurious result.

Although the discrepancy of 5.9 dB between the NF and other was only at one frequency, namely 2000 Hz, and the difference was minimal, this was clinically relevant for speech understanding.

The current findings are consistent with those of Cosetti et al. (2013) who found that electrode type did not have any effect on the hearing findings. In a review paper, Brant and Ruckenstein (2016) conclude that although there is some evidence that indicates that shorter electrodes may improve hearing preservation, studies that directly examine the effect of implant length on hearing preservation in similar patient populations are required.

The large majority (see Table 3) of the participants (83% overall) had the Contour Advance electrode array implanted. Other classifications included ‘other’ electrode types (8% overall).

**TABLE 3:** Electrode array type and insertion in the current sample (*N* = 60).

Current sample (*N* = 60)	Overall %
**Electrode type**	
Contour advance	83
Other	8
Missing	8
**Electrode array insertion**	
Scala tympani	82
Other	5
Missing	13

The Contour Advance electrode is the only perimodiolar array in the industry and is pre-curved for complete insertion, with Softip that enables atraumatic insertion (Roland, Shelva, Gibson, & Treaba, 2005).

As only five non-Contour Advance electrodes were implanted, it would not have been possible to compare the results; thus, the researcher found it to be impractical to perform this analysis which looked at the relationship between the hearing findings and the electrode array type. Therefore, the researcher did not perform this analysis as the data obtained would have no relevance to the findings.

In Skarzynski et al.’s (2002) study, it was found that the electrode type did not have any effect on the hearing findings post-operatively. Furthermore, Dhanasingh and Jolly (2017) did not consider electrode type as one of the key factors that influence hearing performance post-implantation in their overview of cochlear implant electrode array designs.

#### Electrode array insertion

The findings with regard to the percentages for electrode array insertion techniques at the two centres and overall are presented in Table 3. From surgical reports, as this was a retrospective record review, it was evident that the majority of participants had their electrode arrays inserted in the scala tympani during surgery (82% overall). The fact that the current study did not include a review of radiology results for this determination is acknowledged as a limitation and should be taken into consideration when interpreting current findings. The category ‘Other’ included *inter alia* one case involving electrode array insertion via the scala vestibule, possibly because of obstructed scala tympani (Kiefer, Weber, Pfennigdorff, & Von Ilberg, 2000) and another via the round window. However, given the fact that there was only one patient in each case, a meaningful statistical analysis could not be performed on the results; also no significant effects were anticipated because the electrode is placed via the round window into the scala tympani.

The technique used in this study is supported by Adunka, Pillsbury and Kiefer (2006), as 90% of their patients retained their residual hearing post-operatively. In the current study, there was one case where the electrode was inserted in the scala vestibuli. However, review of the surgical notes of this case did not indicate any surgical complications. This was surprising as in previous studies it has been discovered and reported that insertion via the scala vestibuli can result in intracochlear damage (Adunka et al., 2006), as well as variable speech perception outcomes (Lin, Marrinan, Waltzman, & Roland, 2006).

A total of 91% of patients were implanted in the scala tympani and there was a 92% success rate in the preservation of residual hearing (partial and complete) at both centres combined; therefore, it can be inferred that this technique did not negatively influence the hearing findings. O’Connell, Hunter and Wanna (2016) assert about the superior audiologic outcomes for electrode arrays inserted entirely within the scala tympani.

#### Electrode array insertion depth

In the current study (Table 4), the researcher found that the majority of the participants (93%) underwent complete insertion of the electrode array during surgery.

**TABLE 4:** Electrode insertion depth and surgical technique in the current sample (*N* = 60).

Current sample (*N* = 60)	Overall %
**Electrode insertion depth**	
Complete	93
Partial	2
Missing	5
**Surgical technique**	
Contour advance	8
Advance off-stylet (AOS)	67
Other	20
Missing	5

There was only one case of electrode array insertion depth with partial insertion, possibly because of obstructions within the cochlea, such as cochlear otosclerosis, cochlear anomalies and so on (Lee, Nadol, & Eddington, 2011). The remainder of the data consisted of the electrode array depth being complete insertion. As a result of this, the results were not comparable. Thus, the researcher did not perform this analysis as it would not have added any meaningful contribution to the findings.

Nonetheless, current findings where preservation of residual hearing was achieved with full insertion depth for the majority of the cases are supported by Bruce, Bates, Melling, Mawman and Green (2011) who emphasised that the insertion depth was critical to the successful conservation of residual hearing. Numerous other studies reviewed by Miranda, Sampaio, Lopes, Venosa and de Oliveira (2014) revealed that limiting the depth of electrode insertion preserved low-frequency hearing, although this did not include maintenance of ability to discriminate for all patients.

Current findings are inconsistent with what Turner, Gantz, Vidal, Behrens and Henry (2004) have also reported, where they stated that less damage is caused to intracochlear structures by using short electrode arrays rather than the more invasive long electrode arrays. James et al. (2005) studied 12 patients who had received a long electrode and reported that there was preservation of post-operative residual hearing. Contrary to the findings of earlier studies, the post-operative unaided hearing threshold levels in the current study were not affected by the insertion depth.

#### Surgical techniques

The findings for the surgical techniques are summarised in Table 4. Two main surgical techniques were used at the two centres: Contour Advance and the advance off-stylet (AOS) techniques. The majority of surgeons (67% overall; 33% at unit A and 94% at unit B) used the AOS technique.

The majority of surgeons utilised a cochleostomy approach, with the exception of four cases where the round window approach was used. Mangus, Rivas Tsai, Haynes and Roland (2012) assert that the round window approach has advantages for the conservation of residual hearing over the cochleostomy approach that may result in intracochlear trauma. However, the positive findings in the current study seem to dispute this because the cochleostomy approach achieved success in preserving residual hearing post-operatively. Briggs et al. (2006) observed in their study that both approaches successfully avoided trauma to the cochlea during surgery, thus conserving post-operative residual hearing.

The examination of the surgical techniques was considered to be relevant in terms of establishing the possible effect or lack of effect on the preservation of residual hearing. By investigating their effects on the hearing findings, the researcher was interested in determining whether certain surgical techniques were more favourable than others in preserving residual hearing. Thus, an analysis was carried out to establish whether a relationship existed between the hearing findings and the surgical techniques employed.

The effect of surgical technique on change in hearing thresholds was not significant for any of the frequencies, except for 2000 Hz, where the mean change in hearing thresholds was 10.1 (10.3) dB lower for Contour on stylet (Contour) versus AOS, and the mean change in hearing thresholds was 7.9 (6.8) dB higher for other versus AOS. As this discrepancy was limited to one frequency (2000 Hz), and as the change in hearing threshold levels was minimal, this was not statistically significant.

#### Intraoperative complications

From the surgical notes of the surgeons, the researcher investigated surgical complications to determine the possible influence on residual hearing and whether they affected the conservation of functional hearing post-surgery. These are reported in the Gautschi-Mills et al. (2018) study, which included intraoperative complications such as adhesions (3%), drill out of basal turn (3%), Gusher (5%) and trauma (10%), with no complications reported in 68% of the participants.

According to Clark, Tong and Martin (1981) and Balkany et al. (cited in Di Nardo et al., 2007), surgical complications affect anatomical structures following implantation. A relationship between the surgical complications and hearing threshold levels became apparent in the current study where the researcher found a direct correlation between the surgical complications and the hearing findings.

When summarising the relationship between the hearing findings and the various factors listed above, it became evident that the variables investigated had little or no negative effect in general on the preservation of residual hearing, with the exception of surgical complications (Gautschi-Mills et al., 2018). It was found that the aforementioned complications during cochlear implant surgery had a direct influence on the change in residual hearing in that the surgical complications resulted in loss of residual hearing.

## Conclusion

The findings of this study indicated that preservation of residual hearing was successfully achieved at the two centres under consideration. Preservation of post-operative residual hearing was achieved in 92% of participants. An exploration of factors that could have an influence on post-operative preservation of hearing thresholds found that the following variables investigated did not affect the post-operative hearing findings: degree of preoperative hearing loss, aetiology, duration of preoperative hearing loss, as well as the duration between surgery and the unaided post-operative hearing testing. In general, the surgeons in the current study used the same electrode array, insertion method, insertion depth and implant manufacturer. Therefore, comparative statistics could not be performed. From the positive findings of 92% preservation of residual hearing, it could be inferred that optimal results for conservation could be achieved by using the Contour Advance electrode from the manufacturer – Cochlear, the AOS technique and complete insertion of the electrode into the cochlea via the scala tympani – as was performed in the majority of cases in the current study.

Current findings should be interpreted within the identified methodological limitations. Firstly, the retrospective data review nature of this study had its limitations in terms of controlling for possible data collection confounding factors. Secondly, the fact that current findings only looked into two sets of results per participant (one pre-surgery and one post-surgery audiogram) means that the current findings show a snapshot at one point in time post-surgery without establishing whether the preserved residual hearing thresholds remained stable over time. Thirdly, although the current sample size was deemed large enough, the fact that it was conducted in only two cochlear implant units in South Africa limits the generalisability of the findings to the broader population in South Africa. Despite these limitations, the findings have implications for clinical assessment and management, as well as for surgical management of cochlear implant candidates. Preoperative counselling of cochlear implant candidates with residual hearing may benefit from current evidence as candidates may be informed about the possible benefits and risks involved. Future cochlear implant candidates with residual hearing and those candidates for EAS may therefore benefit from these findings as these may assist them in the decision-making process. The conservation of residual hearing post-surgery has many advantages for future cochlear implant candidates with preoperative residual hearing and for those implantees with preserved post-operative residual hearing. These cochlear implant candidates and recipients could then become candidates for EAS, which, in turn, has many hearing benefits, such as improved speech perception in noise and musical acuity. Evidence of the negative impact of intraoperative complications on preservation of residual hearing found in the current study raises implications for surgeons involved in cochlear implantation.

For replication and generalisation of the current findings, as well as opportunity for diversity in the various variables explored, it is recommended that this study should be conducted on a broader population at a number of different cochlear implant centres across South Africa, with larger sample sizes, in order to obtain data that could be generalised to an even broader population in South Africa.
